# Harnessing learning biases is essential for applying social learning in conservation

**DOI:** 10.1007/s00265-016-2238-4

**Published:** 2016-12-07

**Authors:** Alison L. Greggor, Alex Thornton, Nicola S. Clayton

**Affiliations:** 1 Department of Psychology, University of Cambridge, Cambridge, UK; 2 Department of Biological Sciences, Dartmouth College, Hanover, NH USA; 3 Centre for Ecology and Conservation, University of Exeter, Penryn, UK

**Keywords:** Conservation, Environmental change, Learning biases, Social learning

## Abstract

Social learning can influence how animals respond to anthropogenic changes in the environment, determining whether animals survive novel threats and exploit novel resources or produce maladaptive behaviour and contribute to human-wildlife conflict. Predicting where social learning will occur and manipulating its use are, therefore, important in conservation, but doing so is not straightforward. Learning is an inherently biased process that has been shaped by natural selection to prioritize important information and facilitate its efficient uptake. In this regard, social learning is no different from other learning processes because it too is shaped by perceptual filters, attentional biases and learning constraints that can differ between habitats, species, individuals and contexts. The biases that constrain social learning are not understood well enough to accurately predict whether or not social learning will occur in many situations, which limits the effective use of social learning in conservation practice. Nevertheless, we argue that by tapping into the biases that guide the social transmission of information, the conservation applications of social learning could be improved. We explore the conservation areas where social learning is highly relevant and link them to biases in the cues and contexts that shape social information use. The resulting synthesis highlights many promising areas for collaboration between the fields and stresses the importance of systematic reviews of the evidence surrounding social learning practices.

## Introduction

Research generated from the interface of behavioural ecology and comparative cognition can have important conservation implications, not just for those who study species of conservation concern. Issues that involve feeding, space use and survival around predators and anthropogenic threats are at the forefront of conservation behaviour research (Greggor et al. 2016a). As humans profoundly alter the environment, flexible decision making is important for animals in each of these contexts. Regardless of whether this flexibility involves the creation of novel behaviours, such as feeding innovations (Sol et al. 2002), or adjusting existing behaviours in response to changing environmental cues, such as timing migration, species that are able to adjust their behaviour are more likely to survive (Berger-Tal and Saltz 2016a).

One key cognitive process that facilitates flexible behaviour is learning. Where and when animals learn are important to conservation because these can allow animals to acquire appropriate behaviour without having to undergo genetic change (Brown 2013) and can be used as a tool in conservation management (Custance et al. 2002; Whitehead 2010; Greggor et al. 2014b; Schakner and Blumstein 2016). However, learning is still under-utilized in most conservation contexts, including species translocations, invasive species control and human-wildlife conflict (Bell 2016; Berger-Tal et al. 2016). Social learning, i.e. learning from the actions of others or the by-product of others’ behaviour (sensu Heyes 1994; Hoppitt and Laland 2008), can accelerate the spread of behavioural change, with fewer costs than individual learning (Boyd and Richerson 1985). By this definition, social learning occurs in many species ranging from insects to great apes and across contexts ranging from mate choice to foraging and to predator avoidance (Hoppitt and Laland 2013). Because of the importance of these contexts for conservationists, social learning principles are widely relevant to species of conservation concern (Custance et al. 2002; Whitehead 2010). However, despite the overlap of general principles between the two fields, actually using knowledge about social learning can be challenging given the details of conservation interventions.

Imagine, for example, that certain villages are suddenly having problems with endangered species X raiding their soybean crops. Crop raiding is spreading rapidly and threatening the livelihood of people in the villages. There has been support within the communities to cull species X to prevent the destruction of their crops. Should all farmers that grow this crop target species X, even if their farms have not yet been damaged? Or is it a matter of a few problem individuals spreading the behaviour to others? If so, which individuals should be culled and how will their removal influence the behaviour of others? Meanwhile, instead of culling, a local conservation agency suggests employing deterrents to keep species X away from farms. However, one individual of species X in particular is known to have ignored deterrents in the past. Will other individuals learn that deterrents are not dangerous and quickly render them useless? Effective management of this problem is essential to the survival of species X and to the livelihood of the villagers, but effective culling strategies and deterrent methods rely on predictions about whether or not conflict behaviours will be learned socially and spread equally throughout the population.

Even in this simplistic example, the pace of behavioural acquisition is important, as is the potential unevenness of its spread between individuals. In these and many other types of problems that conservation managers face, an understanding of how social learning operates in the wild is crucial for effective conservation practices that involve and target animal behaviour. However, predicting where social learning will occur is not straightforward (Rendell et al. 2011), and this limits our current ability to implement conservation management and policy that utilizes social learning processes. This review will detail the potential conservation uses of social learning and will highlight areas where social learning biases deserve greater attention because of their conservation implications.

## The role of social learning in conservation

In practice, conservation has three main aims: (1) to determine what biodiversity needs to be conserved, (2) to identify and assess threats to biodiversity and (3) to develop solutions that mitigate current or potential biodiversity losses (Groom et al. 2006; Primack 2006; Berger-Tal and Saltz 2016b). Social learning has a role in each of these aims and has therefore been suggested to be important in conservation contexts (Whitehead 2010; Greggor et al. 2014b; Schakner and Blumstein 2016; see Table 1). Meanwhile, there has been progress in recognizing the value of social learning in conservation on the international policy stage. The last meeting of the Convention on Migratory Species (CMS 2014) explicitly discussed the impact that cetacean culture (i.e. socially learned, group-specific behavioural variants) should have on species’ management and policy. However, despite proposing that socially learned behaviour needs to be considered when conserving migratory species, these proceedings only acknowledged a small portion of the potential uses of social learning in conservation. By exploring each of the main conservation aims in more detail, the extent of where social learning could be used in management and policy becomes apparent.

**Table 1:** The use of social learning for each main conservation aim

Conservation aim	(1) Quantify biodiversity	(2) Understand threats to biodiversity		(3) Mitigate threats to biodiversity	
Social learning application	Catalogue socially learned behavioural variants that impact survival	Determine where social transmission is at risk	Predict where animals will be flexible in avoiding threats or adjusting to change	Prevent maladaptive behaviour	Encourage uptake of novel behaviour
Example use	Measure orca group-specific behaviours^a^	Forecast interference in fish chemical communication^b^	Model whether avian migration routes respond to climate change^c^	Stop information spreading about the non-threatening nature of deterrents	Enhance predator avoidance training before release into wild^d^

^a^Ford and Ellis (2006)

^b^Mirza et al. (2009)

^c^Keith and Bull (2016)

^d^Griffin (2004)

### Aim 1: what to conserve?

Social learning can create behavioural variants that form valuable units of biodiversity. When information consistently travels from parents to offspring, social learning can act as a form of non-genetic inheritance (Laland et al. 2001, 2009), which can drive population differences in behaviour. When social learning leads to the adoption of fitness relevant behaviours or drives reproductive isolation in sympatric groups, socially learned behavioural variants can shrink effective population sizes, thereby magnifying threats that would otherwise be considered over a geographical area (Whitehead et al. 2004; Ryan 2006). Additionally, culturally derived behaviours and the conformity they promote can put certain populations at greater risk than others. For example, even though orcas (*Orcinus orca*) are capable of capturing a variety of prey, southern resident populations (a socially and culturally distinct group) mainly hunt chinook salmon (*Oncorhynchus tshawytscha*) (Ford and Ellis 2006) and are therefore more vulnerable than other groups of orcas if salmon populations decline (Whitehead 2010). Finally, it has been argued that some group-specific, socially learned behaviours are worthy of conserving in their own right because their loss would result in unwanted homogenization of species’ behaviours (Slabbekoorn and Smith 2002; Whitehead 2010). For example, the diversity of song in Dupont’s lark (*Chersophilus duponti*) declines as habitat fragmentation increases (Laiolo 2008), a type of loss akin to the disappearance of human languages (Laiolo and Jovani 2007). Thus, quantifying stable, socially learned behaviours can inform conservation management about population vulnerability and behavioural variation worth saving (Whitehead 2010).

### Aim 2: what threatens biodiversity?

Social learning can benefit animals where it helps them adjust to human induced changes in the environment, but it can also prove detrimental where it spreads behaviours that lead to human-wildlife conflict. Therefore, predicting where social learning occurs can be important for assessing threats posed by human activities. In species that rely on the social transfer of information for survival, identifying where human activities could disrupt information transfer is essential for accurate threat forecasting. For example, chemical pollutants such as heavy metals can blunt fishes’ responses to conspecific alarm cues (Mirza et al. 2009), which would reduce their ability to learn socially about predators. Meanwhile, anthropogenic noise can distract attention and mask auditory communication channels (Shannon et al. 2015), such that individuals would be less likely to pick up on alarm vocalizations that could lead to learning about predators (Grade and Sieving 2016). In this way, identifying where survival mostly rely on social learning can help prioritize which species or populations most need protection from processes that interfere with information transfer, such as chemical pollution or anthropogenic noise.

As well as disrupting learning channels, human activity can also reduce the diversity of information carried socially in populations. The loss of socially learned information as a result of population decline can have long-term consequences that threat forecasting should strive to evaluate. For example, it has been suggested that the failure of the North Atlantic right whale (*Eubalaena glacialis*) population to rebound after whaling was banned might be due to the their loss of socially retained information about feeding habitats (Whitehead et al. 2004). Thus, accurate assessments of species recovery rates may need to consider the social learned information that populations carry when determining their relevant Allee effects (i.e. inverse density dependence; fitness-driving processes that are magnified as population size decreases; Stephens et al. 1999).

In a more positive sense, the occurrence of social learning amid human-induced habitat changes can also have net benefits for species survival where it allows animals to flexibly respond to threats. Social learning can help spread novel behavioural variants that facilitate animals’ ability to adjust to environmental changes (van der Post and Hogeweg 2009; Brown 2012; Rubenstein 2016), such as the uptake of innovations (entirely novel behaviours) that allow for the adoption of new foods or foraging techniques (Ramsey et al. 2007). For example, black rats (*Rattus rattus*) in Israel created a new foraging niche and expanded their range into nearby forests by adopting a socially learned foraging technique that allowed them to eat pine cones (Terkel 1996). More generally, foraging innovations have been linked to invasion success, at least in birds (Sol and Lefebvre 2000; Sol et al. 2002). Meanwhile in primates, species-level measures of foraging innovations are correlated with social learning (Reader and Laland 2002). Thus, social learning of novel foraging techniques could more widely accelerate behavioural adjustments to changing habitats, and those species which are most innovative might be most likely to spread novel behaviour. Models that aim to predict the spread of invasive species or the resilience of species to shifting climate could benefit from incorporating this information (Keith and Bull 2016).

The acquisition of novel behaviour and the expansion of niches via social learning may equally serve as a threat to biodiversity when innovations put species in conflict with people. There have been documented cases where social learning has been implicated in aiding the spread of conflict or problematic behaviours, such as the depredation of fishing catches by sperm whales (*Physeter macrocephalus*) (Schakner et al. 2014a), crop raiding behaviours by African elephants (*Loxodonta Africana*) (Chiyo et al. 2012) and spreading bears’ and dolphins’ reliance on anthropogenic foods (Mazur and Seher 2008; Donaldson et al. 2012). These types of conflict can foster negative attitudes towards wildlife, can reduce support for local conservation programs and lead to persecution or culling of conflict species (Mpanduji et al. 2004; Thirgood et al. 2005). Additionally, since social learning does not always spread the most efficient or rewarding behaviour (Boyd and Richerson 1985; Laland and Williams 1998; Giraldeau et al. 2002), animals may socially learn conflict behaviours despite other, equally beneficial options being available. For example, in the case of black bears (*Ursus americanus*), Mazur and Seher (2008) found that cubs obtain preferences for human-developed or natural habitat from their mother, regardless of the presence of both habitat types in the area. Moreover, the bears in developed habitats were 45 times more likely to feed on human-produced food, even though a third of these bears would not survive past their first year due to conflict-related deaths. Therefore, conflict behaviours that spread via social learning can persist even if they reduce fitness. Overall, the likelihood that species will acquire and spread either adaptive or maladaptive behaviours is vitally important to predicting future resilience or conflict with humans.

### Aim 3: mitigating threats to biodiversity

Social learning can be harnessed to enhance or prevent behaviour that would aid conservation goals. Social learning has been proposed to be useful in reducing the incidence of road collisions (Proppe et al. 2016) and has been found to help spread information about novel predators in reintroduction programs (Griffin 2004) and increase the survival of reintroduced hatchery-reared fish (Brown and Laland 2001; Brown and Day 2002). Moreover, some of the detrimental behaviours that animals pick up post release, such as an attraction or tameness towards humans, can be prevented by giving animals time to interact with knowledgeable, adult conspecifics prior to release (Walters et al. 2010). Social learning can also be promoted by broadcasting attractive social cues to help animals colonize newly restored habitat or avoid settling in poor habitat that might otherwise be perceived as high quality, thereby preventing perceptual errors and ecological traps (sensu Gilroy and Sutherland 2007). For instance, social cues have been harnessed, in attracting threatened bird species to settle in suitable habitat by playing conspecific song (Virzi et al. 2012) or erecting conspecific decoy models (Kress and Nettleship 1988).

In contrast, less is known about how to prevent the social transmission of behaviour that may exacerbate conservation problems. Schakner and Blumstein (2016) put forth theoretical tactics for preventing learning that could, in principle, be adapted to social learning situations. They propose that populations can occupy one of several learning stages with regards to a specific behaviour—pre-learning, mid-learning, and post learning—each of which may be targeted with different tactics by conservationists. Before the conflict behaviour has entered the population, mitigation techniques should focus on preventing individual learning about the outcome of the behaviour. For example, in bear populations without conflict, efforts should focus on keeping human food out of reach. If the behaviour has entered the population but has not fully spread—i.e. during association formation—management would benefit from removing individuals that have already acquired the conflict behaviour. If the behaviour is ubiquitous throughout the population, deterrents may be the only viable solution to prevent the behaviour. Future development and testing of such tactics in conservation settings are fundamental to many conservation policies because social learning can easily derail a management solution, like spreading wariness of traps or decreasing the effectiveness of deterrents. For example, it has been suggested that population estimates of sperm whales in the nineteenth century were underestimated because, individuals learned socially to avoid boats (Smith et al. 2008). Had there been a way to reduce or reverse transmission of this fear, populations could have been surveyed more accurately.

Finally, while we only mention it briefly here, similar types of strategies for preventing the transmission of behaviour or encouraging its adoption could also be employed on the human side of conservation. Conservation solutions rely heavily on information exchange between networks of organizations and communities, and many conservation problems, especially in cases of human-wildlife conflict, need to address the root human cause of the biodiversity threat for effective mitigation (Woodroffe et al. 2005). Insight gained from social learning in general could help spread information about conservation initiatives or practices (e.g. sustainable harvesting), or change cultural behaviour (Clayton and Myers 2015), such as reducing the demand for ivory or shark fin soup. In contrast, there are instances where the maintenance of cultural norms, such as taboos on certain hunting practices, are equally as important in conserving species as legislative change (e.g. in Madagascar Jones et al. 2008). Even though encouraging or preventing social learning in humans carries with it necessary logistical and ethical considerations, social learning research that is applicable to humans has great conservation implications.

### Barriers to using social learning

Despite its many potential conservation uses, social learning is not widely used in practice. Even in areas where social learning has been developed as a tool for altering behaviour, such as in reintroduction efforts, prescribed interventions do not exist for many species or contexts. Simply exposing conspecifics to an experienced individual, which is often recommended (e.g. Shier 2016), may not help if individuals differ in their propensity to attend to or learn from others (Mesoudi et al. 2016). Additionally, certain social stimuli may never be learned if they fail to attract attention or do not consistently occur with perceivable outcomes. Although these issues have been commonly researched in the social learning literature (see Rendell et al. 2011), they are not often considered in conservation practice; yet they are crucial to determining the frequency and strength of cues necessary to produce or prevent social learning.

With the explosion of social network-based diffusion analysis—i.e. a technique that identifies social learning based on whether information spreads preferentially between individuals who associate or interact most frequently (Farine and Whitehead 2015)—it is becoming easier to identify behaviours that are learned socially and to identify biases in information spread. The development of these techniques offers an unprecedented opportunity to integrate social learning knowledge into conservation practice. However, social learning is still primarily studied in captivity. Accurately predicting, for example, the influence of social learning on species’ flexibility in their responses to climate change (e.g. Keith and Bull 2016) requires that learning biases are also resolved in the wild if such modelling is to be representative of real world behaviour.

Without clear expectations for social transmission and guidelines to encourage or inhibit the spread of information, socially targeted mitigations are not going to be widely used in conservation. Calls for targeting learning as part of conservation interventions continue to grow (Greggor et al. 2014b; Berger-Tal and Saltz 2016b) and so too does the importance of using social learning effectively. By highlighting many of the biases that are relevant to conservation practice, it becomes clearer where current knowledge about social learning can be better used, and where further research is needed.

## Learning is biased

Animals perceive, remember and act on information from the environment in a biased way. All animals, including ourselves, filter irrelevant details from the information that we perceive and learn about. This filtering process is shaped by natural and sexual selection, such that each species is primed to attend to and learn about the types of information that it most needs to survive and reproduce (Shettleworth 2010). For example, since it has been evolutionarily advantageous for a generalist scavenger like a rat to avoid food that causes illness, rats rapidly associate taste cues with feeling ill, even after a single encounter (Garcia et al. 1974), a process called conditioned taste aversion. However, rats fail to make the same association when a light instead of the taste of food precedes the feeling of illness (i.e. the Garcia effect). In contrast, other species that consume a single food type, such as vampire bats (*Desmodus rotundus*), fail to show taste aversion learning (Ratcliffe et al. 2003). Therefore, not all stimuli are equally likely to cause learning and not all species are equally likely to learn from the same stimuli. The guiding principles of learning predict that stimuli which are more perceptually salient, predictable and biologically relevant are more likely to be learned (Shettleworth 2010). Such learning biases are widespread in the animal kingdom.

Many of these same biases apply when considering social learning because it involves similar associative learning mechanisms, but with attention drawn towards social cues (Heyes 1994, 2012; Dawson et al. 2013; Leadbeater 2015). For example, rhesus monkeys (*Macaca mulatta*) will learn socially to fear snakes, an evolutionarily relevant predator, but will not learn to fear flowers, even if they see demonstrators reacting fearfully towards them (Mineka and Cook 1988; Cook and Mineka 1989). Despite the similarities between social and asocial learning, predicting whether individuals will use social information relies on understanding on an additional layer of biases that guide attention towards the demonstrator. Both vertebrates and invertebrates exhibit social learning biases or “strategies” that can sometimes offer conflicting predictions about “when”, “where”, and “from whom” animals copy information, depending on the species, the context and time of year (Kendal et al. 2005; Rendell et al. 2011; Grüter and Leadbeater 2014; Greggor et al. 2016b). For example, female guppies (*Poecilia reticulata*) only copy older females (Dugatkin and Godin 1993), whereas squirrel monkeys (*Saimiri*) will copy individuals of either sex (Hopper et al. 2013). How then should a manager predict which sex will spread information in a different species?

When scrutinized in this way, certain gaps in our knowledge about social learning highlight how difficult predictions about the social spread of novel behaviour can be. For example, there is lots of evidence that animals learn socially about what, where and how they should eat, especially during development (Galef and Giraldeau 2001; Visalberghi and Addessi 2003; Thornton and McAuliffe 2006; Thornton 2008; Thornton and Clutton-Brock 2011). In contrast, there is less research about how wild animals socially learn about what not to eat when encountering novel foods in adulthood (Galef and Giraldeau 2001), although there is evidence that some species may socially acquire food avoidance (Mason and Reidinger 1982; Snowdon and Boe 2003). Predicting the occurrence of social learning is complicated because biases can differ at several levels: between habitats, between species, between individuals and between the cues that individuals encounter. Each of these types of biases is discussed separately below, but in reality, they are overlapping processes in which several might apply in a given situation. Every case discussed where current theory fails to predict the outcome of these biases offers a prime opportunity for future research that advances both social learning knowledge and conservation aims.

### Habitat driven biases

In theory, social learning should be favoured over individual learning when animals are uncertain or acquiring information individually is costly (Rendell et al. 2011), and should be favoured in environments that are variable enough to promote learning as opposed to genetic adaptation, but not too variable such that learned information is quickly outdated (Laland and Kendal 2003). Additionally, the value and variability of resources have also been suggested to influence greater use of social information when competition is high (Smolla et al. 2015). Even though theories about what conditions should favour social learning have been well explored (see list in Fragaszy and Perry 2003; pg. 34), predicting where social learning will actually occur is challenging (Rendell et al. 2011), especially in the wild. Empirical validation is needed for a range of these theories. As a first step, the theory and modelling about what optimal level of environmental variability most encourages social learning need to be mapped onto the real-world conditions of change that animals currently experience because of human influences.

There have been but a handful of empirical field tests of the hypotheses that social learning should be favoured in conditions of mid-level environmental variability. Wilkinson and Boughman (1999) looked at whether the rate at which feeding patches disappeared—a proxy for environmental variability—predicted species’ use of social information at several communal roosts in three bat species that differed in foraging ecology. They found that they could predict the relative amount that each species followed conspecifics out of the roost based on how often bats foraged unsuccessfully on their own. However, their prediction that sufficiently high patch variability would select against social learning in one of their study species was not supported with the data. Such experiments in other wild systems are central to predicting whether social learning propensities will increase or decrease as conditions change. Tests of the propensity for social learning across urban and rural habitats, for instance, where variability is proposed to differ, would be hugely informative. Such tests will help inform predictions about which environments impacted by humans are most likely to interfere with or generate social learning.

### Species-based biases

Species differ in their propensity to learn. However, species ranging from bumblebees (*Bombus terrestris*) (Worden and Papaj 2005) to chimpanzees (*Pan troglodytes*) (Whiten et al. 1999) are routine social learners. Therefore, social learning is perhaps better predicted by the circumstance in question, rather than by assumptions about the learning capacities of the species involved (Laland and Kendal 2003). Species do not have to be social to learn socially (Wilkinson et al. 2010), but social species may have more opportunities for social learning. Thus, social learning is expected to be more common in social as opposed to solitary species (Roper 1986; Lee 1991; Lefebvre et al. 1996; Lefebvre and Giraldeau 1996; Reader and Lefebvre 2001). However, a species’ social system alone cannot predict the occurrence of social learning (Thornton and McAuliffe 2015; Thornton et al. 2016). There are species that may seem like good candidates for social learning, such as spotted hyenas (*Crocuta crocuta*), that live in social groups and are opportunistic foragers, but for which social learning does not influence the uptake of novel behaviours in foraging tasks (Benson-Amram et al. 2014). Such exceptions are not only interesting from an academic standpoint, but explaining why such exceptions exist is crucial to predicting the use of social learning in lesser studied species of conservation concern.

In some cases, there are easily identifiable reasons why a species may not learn socially. For example, alarm behaviour is less likely to lead to social learning in species where alarms do not reliably predict a high threat of predators (Griffin, 2004). For instance, alarm calling in birds can be triggered by the presence of a predator, but can also result from false alarms about non-predatory intruders or be given by deceptive individuals that gain from distracting conspecifics (Møller 1988). Thus, learning immediately and irreversibly about whatever stimulus is paired with conspecific alarm calling could be detrimental. In contrast, for many fish species, where alarm signals are derived from the chemicals released by injured conspecifics, the alarm always indicates the presences of predators and thus should be more robust to extinction (Griffin 2004).

Even if a species learns socially in one context, it does not mean that it will learn socially in all contexts. For example, Norway rats (*Rattus norvegicus*) learn socially about novel foods (Galef 1982). However, these rats do not learn socially about a novel foraging technique in which individuals dive for molluscs on river beds (Galef 1982), perhaps because the behaviour is harder to observe underwater. Meanwhile, the same is true for vervet monkeys (*Chlorocebus pygerythrus*) that appear not to use social information when learning about a new deterrent, such as recently installed electric fencing (Weingrill et al. 2005), even though they learn socially in novel food contexts (e.g. van de Waal et al. 2013). Investigating what separates the contexts in which a species does and does not learn socially would clearly be important in facilitating or preventing social learning.

Species-level biases are also fundamental to predicting threats and advising conservation policy. Migrating species differ in whether they socially learn their travel route; many baleen whales and bird species do, but sea turtles do not (Scott et al. 2014). Several conservation considerations hinge on whether or not a migratory species learns its route socially, such as: do migration routes represent a cultural variant that needs to be maintained, should the loss of migratory knowledge be included in threat forecasting, and should social learning should be encouraged in reintroductions to aid first migrations (e.g. Urbanek et al. 2005)? Meanwhile, differences in species’ social learning propensities can influence the extent to which a management action persists over time. For example, the effectiveness of an intervention designed to prevent waterfowl from taking handouts from park visitors differed by species, potentially because of differences in social learning between them (Conover 1999). Geese (*Branta canadensis*) and swans (*Cygnus olor*) were conditioned to avoid bread through the application of distasteful chemicals. Even though both showed similar levels of initial avoidance, the geese lost their avoidance faster than the swans once regular bread was available because they forage in larger groups where a greater number of social cues were available to indicate food palatability (Conover 1999). Overall, determining which species are most likely to learn socially in a given context is essential to determining if social learning should be used in its conservation.

### Individual-based biases

Another source of bias in social learning stems from who is observing the socially produced information. Some individuals can have preferences for asocially acquired information, even if the information is outdated or suboptimal (Leadbeater and Florent 2014). The developmental stage, sex or personal experience of the observer can all influence how likely an individual is to copy a demonstrator (Nottebohm 1970; Reader and Laland 2000; van Bergen et al. 2004; Thornton and Malapert 2009). Of these observer-driven biases, those that are dependent on the stage of development are best studied. Such biases influence social learning during critical periods, often to help in the development of evolutionarily appropriate behaviour. The acquisitions of birdsong and mate preference are classic examples where information is learned socially during a sensitive period of development (Nottebohm 1970; Clayton 1989). This means, for example, that birds which do not have social exposure to song or the appropriate parent will develop suboptimal song and mate preferences that can reduce their reproductive prospects. While sensitive periods are utilized in many captive breeding programs, such as encouraging socially mediated migration and habitat choices (Urbanek et al. 2005), the mitigation of social learning interference during critical periods has not often been attempted in the wild. Such efforts are limited by the fact that we lack understanding of what happens after interference is removed (e.g. in the case of anthropogenic noise, Shannon et al. 2015).

Understanding where learning is biased towards certain age groups, for instance, could be crucial to accurately assessing where natural channels of information flow might be disrupted by human activities. For example, older female matriarchs carry social knowledge in elephants (*L. africana*), such that groups with older females have higher fitness and respond better towards predators (McComb et al. 2001, 2011). Since older elephants (including females) have larger tusks, they are preferentially targeted by poachers (Dobson and Poole 1998). Similar social learning biases towards older females have also been found in orca social groups (Brent et al. 2015). Therefore, assessing the impact of this social learning bias is crucial to accurately predicting the threats posed by poachers and the demographic costs that the removal of such individuals from the population would have (Whitehead 2010). Also important to such assessments is whether individuals are flexible in employing different social learning strategies that enable them to learn when to switch to a different demonstrator if their demonstrator becomes unreliable or ceases to exist (discussed in Heyes 2016; Mesoudi et al. 2016).

Other factors during development can also influence the probability of a given individual attending to and learning about social information. For instance, the level and timing of developmental stress that quail (*Coturnix japonica*) experience influences their use of social information (Boogert et al. 2013). Developmental stress can be triggered by inadequate food provisioning (Pravosudov and Kitaysky 2006), and areas with high human impact, such as urban habitat, appear to have lesser quality food as evidenced by the stunted development seen in urban populations (e.g. great tits *Parus major* and blue tits *Cyanistes caeruleus*; Bailly et al. 2016). Therefore, researching whether suboptimal habitat influences individual social learning tendencies may help explain natural individual variation in social information use and could be an important component of predicting the impact of urbanization on animal populations.

Whether an animal has previous experience with a stimulus can also influence whether they learn socially about it. Short-term habituation to non-threatening stimuli, on the order of ∼5 presentations, does not hamper fear conditioning, but long-term habituation, such as lifetime experience with a non-threatening stimulus (e.g. a heterospecific), does (Curio 1988). Other types of experiences can also influence whether individuals use social information. Early life experience with conspecifics can influence individuals’ tendency to learn socially in later life (Chapman et al. 2008). Moreover, the type of social experience an individual has with a stimulus can determine whether learning occurs, although the direction of this effect can depend on the species. For example, individual pigeons do not learn a new feeding technique if they scrounged from others who learned the method (Lefebvre and Helder 1997). In contrast, in some other species, such as meerkats (*Suricata suricatta*) and common marmosets (*Callithrix jacchus*), scrounging experience has been shown to promote social learning (Caldwell and Whiten 2003; Thornton and Malapert 2009). These differences in the effects of scrounging on social learning may be linked both to species-level variation in social tolerance (Caldwell and Whiten 2003) and the extent to which the act of scrounging exposes individuals to the instrumental contingencies of a task (Thornton and Malapert 2009).

Another type of bias driven by individuals stems from who is producing information. Demonstrator bias (otherwise known as “indirect” or “model-based” bias; Rendell et al. 2011) can differ depending on the species. For example, nine-spine sticklebacks, chimpanzees and meerkats are more likely to copy older individuals (Dugatkin and Godin 1993; Thornton and Malapert 2009; Horner et al. 2010), while rats (*R. norvegicus*) do not show copying biases relating to demonstrator’s age (Galef 2009). Moreover, a given species can exhibit multiple demonstrator biases, such as copying older and more knowledgeable conspecifics (Kendal et al. 2015). To complicate matters further, the rules about who to copy can vary depending on an individual’s early life experience. Zebra finches (*Taeniopygia guttata*) that experienced higher levels of developmental stress are less likely to copy their parents than unaffected offspring are (Farine et al. 2015). Current research has not revealed general rules that determine these individual copying biases between species. Indeed, simple rules are unlikely to exist, given the number of biases that can apply. However, the better we can predict where similar assumptions about biases hold across related species or social systems, the better management techniques that target social learning will generalize across contexts.

An understanding of individual-based biases that influence how information spreads socially within wild populations is of great importance in several mitigation settings. Selective removal practices that target an individual after they have committed “problem behaviour” are often ineffective in the long term (Treves and Naughton-Treves 2005). Such practices would be more effective, and considerable time and money saved, if managers could determine ahead of time the probability that problem behaviour would spread, and under what timeframe it might do so. Additionally, management solutions that do not have the capacity to target all individuals in a population could benefit from insight into which individuals tend to spread information in a population. It has been proposed, for example, that road collisions could be reduced by installing invisible fences near roads and fitting shock collars on a subset of the population, in the hopes that avoidance will spread socially (Proppe et al. 2016). Again, much time, energy and investment could be saved if collared individuals could be chosen based on their location in the social network and their likelihood of acting as a “demonstrator” for others.

### Cue-based biases

Certain cue-response pairings are easier to learn than others. Stimuli will be more easily learned the more they (a) are conspicuous, (b) reliably precede an outcome and (c) are evolutionarily relevant (Shettleworth 2010). Social learning will occur more quickly and strongly with increasingly salient cues because they are more likely to attract the attention and emotional reaction of an observer. For example, exposure to a conspecific reacting fearfully is more effective than just broadcasting alarm calls alone (Curio 1988).

Learning is also based on contingency. If two cues do not reliably coincide, animals are not likely to learn socially about them. For example, even in species that can learn socially, social learning may not occur in the wild if animals never observe the behaviour they are expected to learn. For example, the planigale (*Planigale maculate*) is a small Australian mammal that is threatened because it consumes a toxic, invasive predator, the cane toad (*Rhinella marina*). Although planigales are capable of avoiding toads through taste aversion learning, and are likely capable of learning socially, there is no evidence that they socially learn to avoid toads (Box and Gibson 2006; Webb et al. 2008; Llewelyn et al. 2010). Presumably, all knowledgeable individuals that individually learn to avoid the toads do not produce social information about toads because they avoid interacting with them. So, unless species commonly forage in groups where they could observe “disgust” responses (e.g. Mason 1988), they are not likely to learn socially in the wild about novel, poisonous food. Predicting the frequency of social information that individuals are likely to encounter is, therefore, an integral part of predicting the spread of socially learned behaviour.

Finally, the ecological relevance of stimuli is crucial for determining whether it will spread socially. In the case of socially acquired predator avoidance, fear-relevant stimuli are more likely to be learned than fear-irrelevant stimuli because the process is adaptively biased to facilitate escape from danger but to limit the amount of time wasted on false alarms (Griffin 2004). Therefore, the information gathered during fear conditioning tends to be content, not context specific, such that animals respond any time they encounter that fear stimulus, irrespective of location (although some species do generalize their socially learned threat responses if they occupy risky environments; Ferrari et al. 2009). It is not always easy to predict what types of stimuli can be associated with predatory threats, since individuals have been shown to socially acquire fears of inanimate objects, such as a coloured plastic bottle, or of non-predatory species, such as goldfish, although not always as strongly as natural fear stimuli (Curio 1988; Chivers and Smith 1994).

## Conservation applications that target social learning biases

Given how many biases can influence social learning, is it wise to advise conservation managers to simply expose individuals to conspecifics if they want to increase a behaviour and limit their exposure if they want to prevent a behaviour from spreading? This is the tactic often employed or suggested (Shier 2016), but crucial details about the species, context and cues could obfuscate the outcomes, even if their social system allowed such manipulations. By accruing many examples of biases and highlighting places for future development, this paper is not meant to imply that social learning is too variable to be used. It does mean, however, that there are dangers with relying on studies from a single species or context to guide the development of novel management protocols in other species or contexts.

Although the field is a long way from developing relevant and useful guidelines for all species and situations, there has been progress in the development of techniques in some areas, such as species translocations that train animals socially about appropriate behaviours prior to release (e.g. Walters et al. 2010). Augmenting these existing practices with insights generated from research on social learning biases will only strengthen their effectiveness. Additionally, there are areas where social learning biases are known, but techniques targeting them have not yet been employed in conservation practice, for instance, in mitigating social learning interference of chemical or auditory alarm cues in areas where noise or chemical pollution is known to interfere with learning channels. These areas represent fertile opportunities for experts in social learning to use their knowledge to shape research and consult with conservationists in cases where they could help solve specific problems. Despite these promising developments, however, there are also high priority areas where more fundamental research is still needed before conservationists can broadly use social learning to predict and mitigate threats, such as the spread of human-wildlife conflict behaviours.

One way of identifying such holes in the literature is by conducting systematic reviews and maps of social learning in conservation practice. Evidence-based systematic reviews bridge the gap between academics and managers by presenting a weighted overview of all studies relating to a topic, including information from the “grey” literature where conservation management outcomes often are reported (Pullin et al. 2007). Systematic maps operate on a similar premise but aim to outline existing knowledge gaps (Haddaway et al. 2016). These types of evidence-based reviews are gaining momentum (Sutherland et al. 2004; Pullin et al. 2007; Walsh et al. 2014) and deserve to be employed with behavioural interventions in mind (Greggor et al. 2014a, 2016a; Schakner et al. 2014b). Not only will such reviews direct managers in the use of existing social learning interventions but also will help prioritize areas where future studies of social learning could be most influential in management contexts. Conservationists are only likely to adopt animal social learning tactics when managers can be provided with evidence that it improves outcomes over existing methods.

## Conclusions

Social learning has many potential conservation applications but is biased by attention, experience and species-level constraints. Understanding social learning biases will be crucial to its effective use in quantifying biodiversity, as a predictive tool to gauge and quantify threats to wildlife and as an active tool for conservation management. It is no surprise that it is rarely used in conservation management, despite its potential, because no clear guidelines exist for tapping into social learning rules. Simply because social learning occurs in one species, individual or context does not mean it will in all others. While these details may be academically interesting, they are a barrier to simple conservation solutions. The field greatly needs systematic reviews to help locate areas where existing evidence strongly supports the use of social learning and to make such evidence readily available. Without evidence, the effectiveness of social learning interventions cannot be compared to traditional methods. Meanwhile, many areas where we do not yet have clear predictions about where, when or why social learning occurs are prime topics for future research by behavioural ecologists and comparative psychologists with conservation in mind. Not only the further study of social learning biases will enrich the behavioural ecology field but also the development of extractable rules and predictions will be of great use to conservation practitioners.
